# Tailoring Interventions for Control of Endemic Carbapenem-Resistant *Acinetobacter baumannii*: An Interrupted Time Series Analysis

**DOI:** 10.1093/ofid/ofae301

**Published:** 2024-05-28

**Authors:** Vered Schechner, Adi Cohen, Yehuda Carmeli

**Affiliations:** National Institute for Antibiotic Resistance and Infection Control, Ministry of Health, Tel Aviv, Israel; School of Public Health, Faculty of Medicine, Tel Aviv University, Tel Aviv, Israel; National Institute for Antibiotic Resistance and Infection Control, Ministry of Health, Tel Aviv, Israel; National Institute for Antibiotic Resistance and Infection Control, Ministry of Health, Tel Aviv, Israel; School of Public Health, Faculty of Medicine, Tel Aviv University, Tel Aviv, Israel

**Keywords:** antibiotic resistance, carbapenem-resistant *Acinetobacter baumannii*, hospital-acquired infections, infection control, interrupted time series analysis

## Abstract

**Background:**

We examined temporal trends in carbapenem-resistant *Acinetobacter baumannii* (CRAB) infections in a hospital with hyperendemic CRAB and assessed the efficacy of varied infection control strategies in different ward types.

**Methods:**

We retrospectively analyzed all CRAB clinical samples from 2006 to 2019 and categorized infections as hospital-onset (HO) or community-onset. We used interrupted time series analysis to assess the impact of interventions on the incidence of all HO-CRAB infections and bloodstream infections (BSIs) at the hospital and ward group levels.

**Results:**

Over 14 years, 4009 CRAB infections were identified (89.7% HO), with 813 CRAB BSI (93.2% HO). The incidence per 100 000 patient-days of CRAB infections peaked in 2008 at 79.1, and that of CRAB BSI peaked in 2010 at 16.2. These rates decreased by two-thirds by 2019. In the general intensive care unit (ICU), hand hygiene and environmental cleaning interventions were followed by a significant reduction in the level of HO-CRAB infections, with an additional decrease in the slope after the introduction of active surveillance and 2% chlorhexidine bathing. In the surgical ICU and surgical department, a reduction in slope or level of CRAB infection was observed after moving ventilated patients to single rooms. In medical wards, a multimodal intervention was followed by a reduction in the slope of HO-CRAB infections and BSIs. In wards where CRAB infections were uncommon, the incidence of HO-CRAB infections decreased throughout the study period.

**Conclusions:**

Ward-specific variables determine the success of interventions in reducing CRAB infections; therefore, interventions should be tailored to each setting.

## Teaser

In this single center interrupted time series analysis, we showed that hospital-wide carbapenem-resistant *Acinetobacter baumannii* (CRAB) hyperendemicity is reversible. Interventions to reduce CRAB infections should be tailored to the specific requirements of patients and units.

## INTRODUCTION

Infections caused by multidrug-resistant (MDR) *Acinetobacter baumannii,* particularly those resistant to carbapenem antibiotics, pose a significant risk to patients. These bacteria are acquired almost exclusively within health care facilities and cause infections mainly in mechanically ventilated and critically ill patients [1]. Managing such infections is challenging due to limited antibiotic treatment options, leading to unfavorable clinical outcomes and increased mortality [2]. The World Health Organization (WHO) has ranked carbapenem-resistant *A. baumannii* (CRAB) in the “critical” category in its global list of priority pathogens [3].

Our hospital experienced the emergence of MDR *A. baumannii,* which led to endemicity in the early 2000s [4, 5]. In the years 2013 to 2019, among blood isolates, the percentage of *A. baumannii* nonsusceptible to carbapenems ranged from 74% to 93% (Ministry of Health, unpublished data). The main sequence types (STs) of CRAB were ST2 and ST3, with multiple clones within ST2 [6, 7]. Before 2017, CRAB screening was not conducted routinely. Several focused studies in our hospital (2015–2016) revealed high environmental contamination by CRAB in the intensive care unit (ICU) and step-up rooms [8].

A comprehensive infection prevention and control strategy, combining horizontal measures like hand hygiene and environmental cleaning with vertical (pathogen-specific) interventions such as active screening and patient isolation, is commonly employed to combat CRAB [9]. However, once CRAB has reached a state of endemicity, it is very difficult to control its spread. The transmission of CRAB within health care facilities and the associated infection risks are influenced by patient-related factors (eg, mechanical ventilation and illness severity) and other factors (eg, unit layout, nursing staffing levels, and adherence to hand hygiene practices). Consequently, the choice of infection control measures may need to be tailored to different ward types and patient populations. We hypothesized that in high-prevalence settings, a one-size-fits-all approach may yield limited success in containing CRAB, and it is thereby imperative to customize interventions to specific hospital settings.

This study aimed to analyze temporal trends in hospital-onset CRAB infections across various hospital wards and to investigate the impact of different infection control interventions on the incidence of all CRAB infections and on bloodstream infections (BSIs).

## METHODS

### Study Design, Setting, and Participants

This study was conducted at Tel Aviv Sourasky Medical Center (TASMC), a tertiary care facility in Israel with 1450 beds, comprising 4 distinct hospitals: an adult general hospital, a children's hospital, a maternity and women's hospital, and a rehabilitation hospital. The adult general hospital has a general ICU (18 beds, all in single-patient rooms) and a surgical ICU (7 beds, in an open space, separated by curtains [2006–2014] and later 11 beds, in single-patient rooms [2014–2019]). Additionally, some non–intensive care wards include step-up rooms (9 medical, 1 surgical) that provide care for mechanically ventilated patients and other patients requiring close monitoring. Each of the 9 medical step-up rooms (1 per ward) accommodates 6 to 7 beds separated by curtains. The surgical step-up room features 4 beds separated by curtains. In other patient care areas, the predominant setup consists of multipatient rooms with 2–3 beds, each separated by curtains, and each room is equipped with its own bathroom. The average occupancy rate was 95%.

The hospital's infection control program is overseen by the epidemiology unit, which was established in 2004 and composed of infection control nurses (4–5 nurses until 2014 and 8 nurses by 2017), data scientists, and dedicated infection control physicians. The program is supported by the microbiology lab.

This study was an interrupted time series analysis (ITSA). We divided the hospital into 5 ward groups: general ICU, surgical ICU, internal medicine wards, surgical department (including all adult surgical wards), and other wards. We included all clinical samples positive for CRAB between January 2006 and December 2019. We excluded samples marked as surveillance cultures by the ordering physician.

### Definitions and Outcome

CRAB was defined as the isolation of *A. baumannii*, *A. baumannii* complex, *A. pitti,* or *A. nosocomialis* resistant to imipenem or meropenem (minimum inhibistory concentration ≥8). We defined a clinical infection as detection of CRAB in a clinical sample, without regard to signs or symptoms. The outcome measures were all CRAB infections and CRAB BSIs. An incident case of CRAB infection was defined as a patient's first positive clinical CRAB culture per hospitalization. An incident case of CRAB BSI was defined as the patient's first positive blood culture for CRAB per hospitalization (even if the patient had CRAB detected earlier in this hospitalization at a different clinical site). Each incident CRAB infection was classified as hospital-onset (HO) or community-onset (CO). Infections were considered HO if the initial isolation occurred on hospital day 4 or later or if detected before day 4 in a patient who had been discharged from a previous hospitalization in the past 30 days. Infections were categorized as CO if detected in the first 3 days of hospitalization, with no hospitalization in the preceding 30 days.

### Microbiological Methods

Isolates were identified using the VITEK 2 or VITEK MS system (bioMérieux, Marcy I'Etoile, France). Susceptibility testing was performed in accordance with Clinical & Laboratory Standards Institute guidelines using VITEK 2 (bioMérieux, France).

### Data Collection

Data were obtained from the hospital's admission, transfer, and discharge (ATD) records and the electronic database of the hospital's microbiology laboratory. Information available for each sample included patient ID, collection date, sending ward, anatomic source, and carbapenem susceptibility. Data on patient-days were obtained from the Ministry of Health [10].

### Basic Infection Control Measures

Basic infection control measures were implemented in all wards from the start of the study period and maintained throughout. These measures included continuous initiatives to disseminate knowledge, prospective surveillance of CRAB acquisitions with feedback (by monthly reports to each ward), and targeted responses in wards experiencing a high incidence of CRAB infections. Additionally, there was ongoing horizontal activity aimed at preventing hospital-acquired infections, such as hand hygiene audits and education regarding use of invasive devices.

Alcohol-based hand rub (ABHR) was available at each bed, as well as in other treatment areas, and staff received instructions to adhere to the “5 moments” hand hygiene guidelines. Cleaning responsibilities were shared between the cleaning staff and nursing aides. A sodium hypochlorite–based disinfectant at a concentration of 1000 ppm (increased to 2000 ppm in step-up rooms) was used. Objects that could not be cleaned with sodium hypochlorite, as per the manufacturer's instructions, were cleaned using alcohol wipes. Routine screening for carriage of CRAB was not performed. Patients with CRAB were placed under contact precautions, with a daily confirmation of contact precaution status in the electronic medical record, although often they were not isolated in single-patient rooms or cohort rooms due to limited availability. Critically ill patients, debilitated patients, and carriers of multidrug-resistant organisms (MDROs) were bathed daily with antiseptic soap containing 4% chlorhexidine. Other patients used standard cosmetic soap. Use of certain broad-spectrum antibiotics required approval by an infectious disease physician.

### Bundle of Interventions

**Figure 1.**
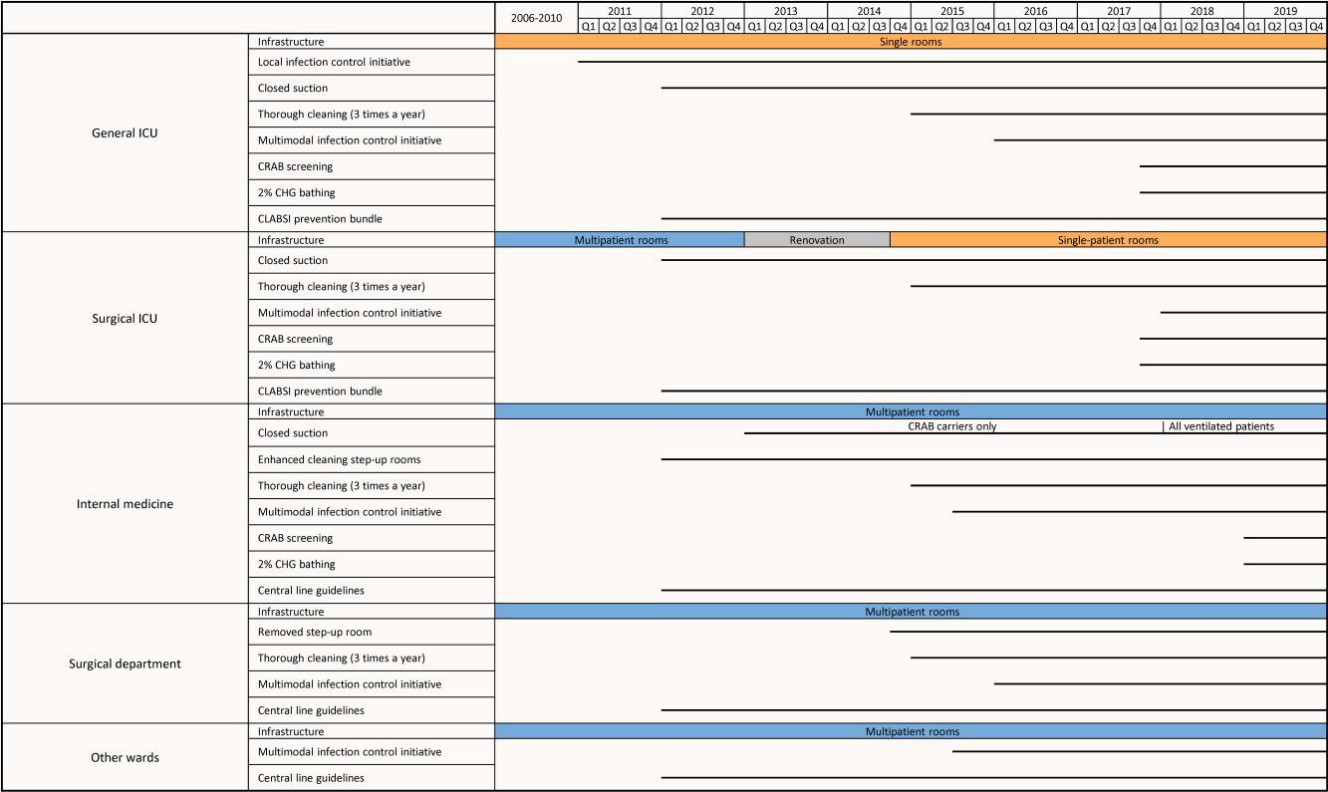
Timeline of interventions, by ward group. Horizontal lines indicate the timing of the interventions. Abbreviations: CHG, chlorhexidine; CLABSI, central line–associated bloodstream infection; CRAB, carbapenem-resistant *A. baumannii*; ICU, intensive care unit.

Different interventions were implemented in different wards at different times, as summarized in Figure 1. Details of specific interventions were as follows:

#### Infrastructure

In 2013, the surgical ICU and the surgical ward housing the step-up room were temporarily relocated for an infrastructure renovation. During renovation, the 4 step-up surgical beds were integrated into the surgical ICU, and the surgical ICU (now 11 beds) was converted into single-patient rooms. The surgical ICU returned to its renovated original location in October 2014.

#### Ward-Level and Hospital-Wide Infection Control Initiatives

The infection control initiative in the general ICU (in 2011) was championed by ward leaders and supported by the hospital epidemiology unit. This initiative primarily centered on enhancing hand hygiene and cleaning procedures. Active staff engagement was encouraged in order to identify challenges and work collaboratively to find solutions.

In July 2015, a hospital-wide multimodal intervention program was initiated, jointly led by hospital management and the epidemiology unit. The program featured a 9-month intervention period for each ward, with 4–6 wards simultaneously recruited. Each ward's intervention was overseen by a team of ward champions, a representative from the administration, and an infection control nurse and physician and actively involved the departmental staff in infection control. The program addressed hand hygiene, environmental cleaning, management of MDROs, BSI prevention, and urinary tract infection prevention. Implementation strategies included training sessions, audit forms, templates for investigating health care–associated infections, and a user-friendly tablet-based system for data collection and tracking. Weekly meetings were held to review audits, incident infections, and root-cause investigation findings. Performance evaluations were presented to each ward quarterly and at the end of the intervention.

#### Environmental Cleaning Enhancements

Environmental cleaning improvements were implemented gradually. In 2012, a biweekly enhanced cleaning protocol was introduced in the step-up rooms, involving curtain changes and cleaning of all objects. Step-up rooms remained occupied during cleaning. In 2015, an additional cleaning initiative was launched, covering the ICUs, internal medicine, and the surgical department. Three times a year, a dedicated team performed a thorough cleaning of the entire department over 2–3 days.

#### CRAB Screening

Beginning in October 2017, a CRAB screening protocol was introduced for all patients admitted to the general ICU and surgical ICU. Patients were screened from the rectum and pharynx upon admission and weekly thereafter. In 2019, the same protocol was extended to internal medicine step-up rooms.

#### Two Percent CHG Bathing

Starting in October 2017, all patients in the general and surgical ICUs received daily 2% chlorhexidine (CHG) bathing. This practice was implemented in the medical step-up rooms in January 2019.

#### Central Line Guidelines and CLABSI Prevention Bundle

In 2012, national guidelines for preventing central line–associated bloodstream infections (CLABSI) were disseminated throughout the hospital. Additionally, mandatory national surveillance for health care–associated BSI and CLABSI was initiated in the general and surgical ICUs, with biannual feedback on infection rates [11]. Between 2012 and 2019, CLABSI cases per 1000 line-days decreased from 7.5 to 2.6 in the general ICU and from 10.0 to 3.6 in the surgical ICU [12].

#### Antibiotic Stewardship

In 2012, an antimicrobial stewardship committee was established, and mandatory national reporting of antibiotic use was initiated. At our hospital, there was no major decline in the use of broad-spectrum antibiotics during the study period [13].

### Statistical Analysis

Incidence rates of CRAB infections (all and BSI) were calculated at the hospital level, divided into HO and CO, and expressed as cases per 100 000 patient-days, per year. Incidence rates of HO-CRAB infections (all and BSI) were calculated at the ward group level and expressed as cases per 100 000 patient-days, per year. An ITSA was performed to evaluate the influence of various interventions on both the magnitude and trajectory of HO-CRAB infections. The analysis employed a generalized least squares regression model, accounting for autocorrelation using a Newey-West standard error correction. We conducted 2 separate analyses, 1 on all HO-CRAB infections and 1 on HO-CRAB BSI only. Incidence rates were calculated at the ward group level and expressed as cases per 100 000 patient-days, per quarter. For the ITSA, we clustered interventions that occurred within 1 year in each ward group and prespecified the knots (defining before and after) at the first quarter of each cluster of interventions. A *P* value of <.05 was considered statistically significant. All analyses were conducted using the R programming language, version 4.3.1.

## RESULTS

### Descriptive Epidemiology

**Figure 2.**
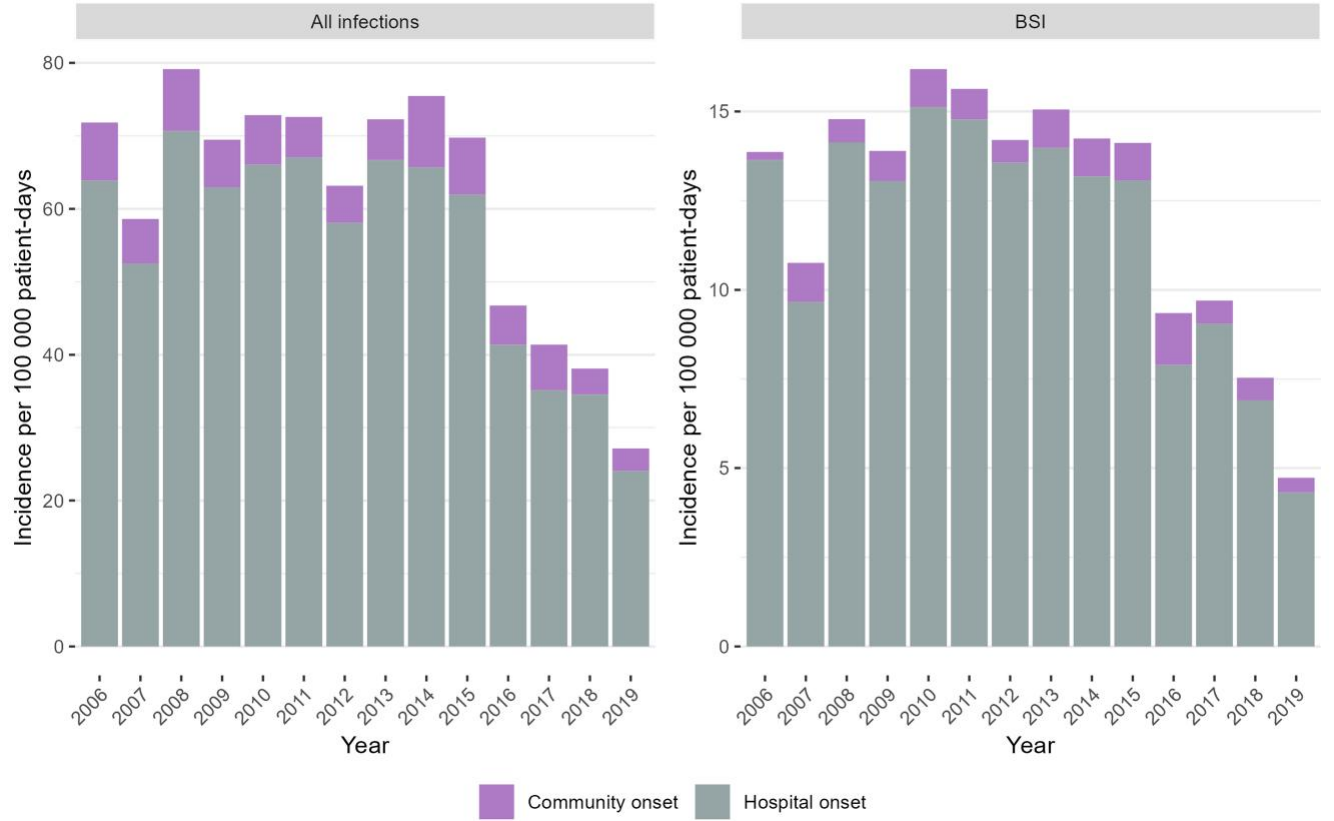
Incidence of carbapenem-resistant *A. baumannii* infections, by place of onset. Abbreviation: BSI, bloodstream infection.

During the 14 study years, there were 4009 CRAB infections and 813 CRAB BSIs. Among the 4009 CRAB infections, 411 (10.3%) were CO and 3598 (89.7%) were HO. These 4009 CRAB infections corresponded to 4148 clinical samples (including multiple positive sources from the same patient, at the time of the first CRAB detection). The sources of the clinical samples were sputum (n = 2037, 49%), urine (n = 533, 13%), blood (n = 471, 11%), cerebrospinal fluid (n = 22, 1%), and other (n = 1,085, 26%). Among the 813 CRAB BSIs, 55 (6.8%) were CO and 758 (93.2%) were HO. The rates of all CRAB infections and BSIs only are presented in Figure 2. The total infection rate peaked in 2008 at 79.1 per 100 000 patient-days and decreased by 66% in 2019 to 27.1. The CRAB BSI rate reached its peak in 2010 at 16.2 per 100 000 patient-days and decreased by 70% in 2019 to 4.7.

**Figure 3.**
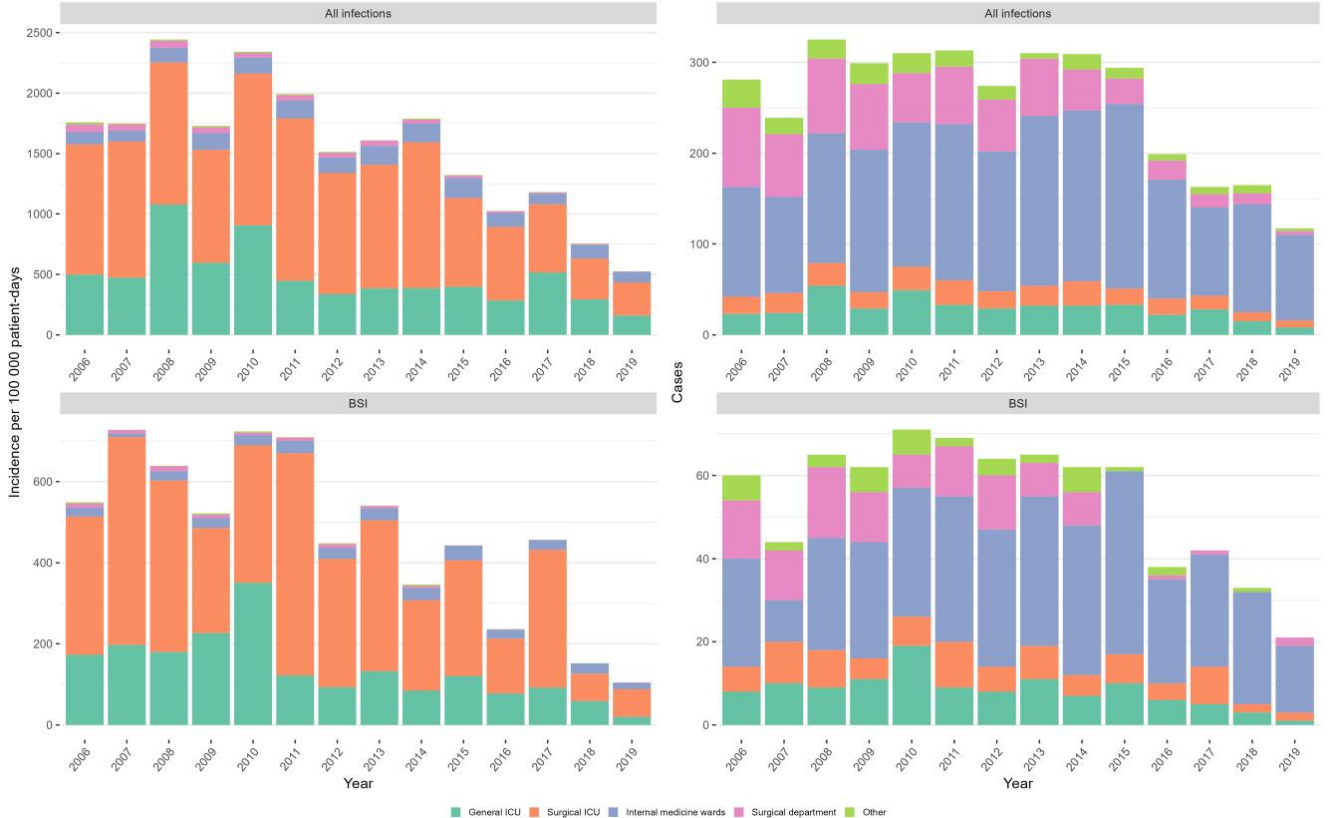
Incidence (left) and absolute number (right) of hospital-onset carbapenem-resistant *A. baumannii* infections, by ward group. Abbreviations: BSI, bloodstream infection; ICU, intensive care unit.

The rates and numbers of HO-CRAB infections and HO-CRAB BSIs, by ward type, are presented in Figure 3. Throughout the study period, HO-CRAB rates were highest in the surgical ICU, followed by the general ICU. The internal medicine wards ranked third, with total CRAB infection rates being less than half of those in the ICUs. Rates in the surgical department and other wards were lowest. While CRAB infection rates were lower in medical wards compared with the ICUs, the absolute number of CRAB infections was highest in these large wards: 2031/3598 (56.5%) HO-CRAB infections and 401/758 (52.9%) HO-CRAB BSIs occurred in medical wards, compared with 685/3598 (19.0%) and 208/758 (27.4%) in the ICUs.

### Impact of Ward Group–Specific Infection Control Measures

**Figure 4.**
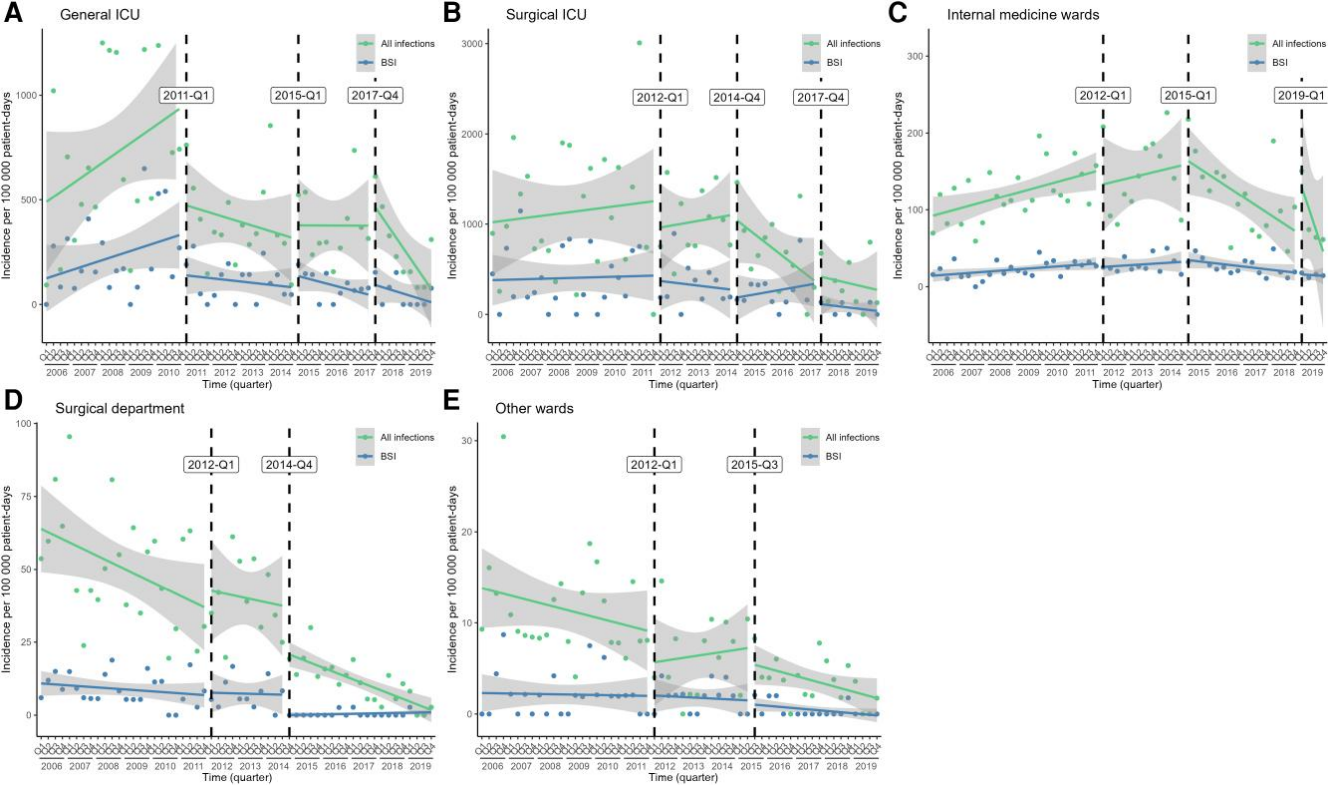
Unadjusted interrupted time series analysis of the effect of different interventions on the incidence of all HO-CRAB infections and HO-CRAB BSIs, by ward group. A—general ICU; B—surgical ICU; C—internal medicine wards; D—surgical department; E—other wards. Abbreviations: BSI, bloodstream infection; HO-CRAB, hospital-onset carbapenem-resistant *A. baumannii*; ICU, intensive care unit.

The results of the ITSA are shown in Figure 4 and Table 1. In the general ICU, there was a drop in the level of both HO-CRAB infections and BSIs (the latter did not reach statistical significance) following the implementation of the ward-level infection control initiative in 2011. After these drops in level, the rates of all infections and BSIs decreased nonsignificantly (relative to the pre-intervention period). There was also a nonsignificant decline in the slope of HO-CRAB infections after the introduction of CRAB screening and 2% CHG bathing (Q4/2017).

**Table 1.** Unadjusted Interrupted Time-Series Analysis of the Effect of Different Interventions on the Incidence of all HO-CRAB Infections and HO-CRAB BSI: Numeric Results

Ward Group	Intervention Point	All CRAB Infections						CRAB BSI					
		Pre-intervention Slope		Level Change		Slope Change^a^		Pre-intervention Slope		Level Change		Slope Change^a^	
		Estimate (95% CI)	*P*	Estimate (95% CI)	*P*	Estimate (95% CI)	*P*	Estimate (95% CI)	*P*	Estimate (95% CI)	*P*	Estimate (95% CI)	*P*
General ICU	Q1/2011	23.4 (−3.5–50.3)	.09	−451.7 (−816.6 to −86.8)	.02	−33.6 (−70.5–3.2)	.07	10.8 (−2.7–24.4)	.12	−188.5 (−396.3–19.3)	.07	−14.6 (−31.1–2.0)	.08
	Q1/2015	…		58.3 (−244.9–361.4)	.70	10.1 (−29.8–49.9)	.61	…	…	61.6 (−39.3–162.6)	.23	−5.0 (−17.5–7.5)	.42
	Q4/2017	…		137.1 (−141.9–416.1)	.33	−48.7 (−100.7–3.3)	.07	…	…	55.9 (−52.5–164.3)	.30	−1.5 (−20.0–17.0)	.87
Surgical ICU	Q1/2012	10.1 (−40.4–60.6)	.69	−304.5 (−1253.2–644.1)	.52	3.5 (−86.8–93.7)	.94	2.2 (−19.0–23.3)	.84	−50.0 (−522.1–422.2)	.83	−11.5 (−60.4–37.3)	.64
	Q4/2014	…		3.2 (−566.4–572.8)	.99	−73.9 (−175.1–27.2)	.15	…	…	−103.0 (−306.4–100.4)	.31	23.1 (−30.7–76.9)	.39
	Q4/2017	…		59.8 (−551.9–671.6)	.84	42.3 (−61.9–146.4)	.42	…	…	−214.9 (−497.4–67.6)	.13	−23.1 (−57.4–11.3)	.18
Internal medicine	Q1/2012	2.5 (0.8–4.2)	.00	−19.2 (−97.6–59.3)	.63	−0.3 (−11.0–10.4)	.95	0.7 (0.2–1.2)	.01	−5.0 (−16.2–6.2)	.38	−0.1 (−2.0–1.8)	.92
	Q1/2015	…		11.8 (−63.7–87.4)	.75	−8.3 (−19.8–3.2)	.15	…	…	3.4 (−14.2–21.0)	.70	−1.7 (−3.8–0.5)	.13
	Q1/2019	…		84.6 (11.2–158.0)	.02	−21.8 (−40.7 to −2.9)	.02	…	…	−1.8 (−14.8–11.1)	.78	0.4 (−1.5–2.4)	.64
Surgical department	Q1/2012	−1.2 (−2.2 to −0.1)	.03	6.2 (−15.5–27.9)	.57	0.7 (−1.8–3.1)	.59	−0.2 (−0.5–0.1)	.26	0.9 (−6.9–8.6)	.82	0.1 (−0.9–1.1)	.83
	Q4/2014	…		−15.8 (−28.5 to −3.1)	.02	−0.4 (−2.7–1.8)	.70	…	…	−7.0 (−12.8 to −1.2)	.02	0.1 (−0.8–1.0)	.81
Other wards	Q1/2012	−0.2 (−0.5–0.1)	.23	−3.6 (−10.3–3.1)	.29	0.3 (−0.4–1.0)	.36	0.0 (−0.2–0.1)	.86	0.1 (−2.5 −2.7)	.95	0.0 (−0.3–0.2)	.84
	Q3/2015	…		−1.6 (−6.0–2.8)	.46	−0.3 (−1.0–0.3)	.30	…	…	−0.4 (−2.3–1.5)	.68	0.0 (−0.3–0.2)	.81

Abbreviations: BSI, bloodstream infection; CRAB, carbapenem-resistant *A. baumannii*; HO-CRAB, hospital-onset carbapenem-resistant *A. baumannii*; ICU, intensive care unit.

^a^Slope after intervention, relative to the pre-intervention period.

In the surgical ICU, the incidence of HO-CRAB infections remained relatively stable until Q3/2014. A nonsignificant negative slope change in the rate of HO-CRAB infections was noticed following the second cluster of interventions in late 2014 (including transition to single-patient rooms and improved environmental cleaning).

In the internal medicine wards, the incidence of HO-CRAB infections, both total and BSI, was rising until 2015. The slope change was negative (although not statistically significant) after the cluster of interventions in 2015 (environmental cleaning and multimodal infection control intervention). The level of HO-CRAB infections increased in Q1/2019, but the slope change decreased significantly after the introduction of active screening and no-rinse bathing.

In the surgical department, the incidence of HO-CRAB infections has been decreasing significantly since 2006. Following the intervention in Q4/2014, which primarily focused on infrastructure improvements, there was a significant reduction in the level of both HO-CRAB infection and HO-CRAB BSI rates, followed by a continued decrease in the slope of all infections but not BSIs (which were rare—only 4 BSIs between 2015 and 2019).

In the other wards, where CRAB infections and BSIs were uncommon, the incidence of HO-CRAB infections (all and BSI) decreased steadily, such that neither of the 2 types of infections defined a clear before-and-after period.

## DISCUSSION

CRAB spread in Israeli hospitals in the late 1990s and early 2000s [4]. At TASMC, CRAB became endemic, with multiple polyclonal outbreaks suggesting both within- and between-ward transmission [5, 14]. In this study, we showed that the high incidence of total HO-CRAB infections and HO-CRAB BSIs persisted from 2006 to 2015. We observed a dramatic decrease in hospital-wide incidence from 2016 to 2019. While there are numerous reports on the control of CRAB in a single affected unit, this study is among the few reports addressing the curtailing of CRAB spread in a setting where it has been prevalent hospital-wide for an extended period.

Among the latter type of studies, Rodriguez-Bano et al. demonstrated the effectiveness of a multifaceted infection control bundle in reducing CRAB incidence, incorporating enhanced infection control measures, active surveillance, strict contact isolation of carriers, and environmental cleaning [15]. Other reports have underscored different components, including communication with wards and data dissemination [16], patient cohorting with dedicated staff [17], antimicrobial stewardship [18, 19], and even temporary hospital closure [20]. Studies highlighting the central role of the ICU as a continuous source of hospital-wide CRAB dissemination showed that CRAB hyperendemicity could be controlled by focusing on the ICU [18, 21].

In our study, while the overall hospital-wide incidence dropped from 2016 onwards, examining the change in incidence by ward group reveals a more complex picture. In each ward group, incidence declined at different time points, and different interventions were effective in each setting. We found that in wards with baseline low incidence of CRAB infections and without ventilated patients, incidence continued to decrease using basic infection control measures and contact precautions. However, in high-incidence intensive care units and wards accommodating ventilated patients, there was a significant reduction in CRAB infections only after implementation of additional measures, including an enhanced isolation infrastructure, environmental contamination control, and active surveillance and 2% CHG bathing. Moreover, the integration of multifaceted infection control interventions and engagement of department champions as partners in the process emerged as important components in this endeavor.

The persistence of *Acinetobacter* in the environment presents a significant challenge, as supported by a study demonstrating that, even after terminal cleaning with bleach, contamination still remained in 44% of rooms after 2 rounds of cleaning and 16% after 4 rounds [22]. This issue becomes even more complex in multipatient rooms, especially those housing ventilated patients, as these rooms are rarely vacant, making complete terminal cleaning often unfeasible. We have previously observed difficulties in eradicating environmental CRAB contamination in step-up rooms [8]. One approach to tackle this challenge is to minimize environmental contamination by implementing closed endotracheal suction systems. However, the adoption of closed suction systems does not provide a complete solution. For instance, 1 study revealed that closed suction systems were unable to reduce acquisition of gram-negative bacteria among ICU patients [23], suggesting that there may be additional sources of transmission, such as nonrespiratory CRAB carriers [24] and non-suction-related activities [25], contributing to environmental contamination. Therefore, to effectively address the challenge of environmental contamination by CRAB, the use of closed suction systems should be complemented by additional measures. Our findings support this concept. Rates of HO-CRAB in the surgical ICU remained high after the introduction of a closed suction system, while a decline in slope was achieved only following the transition to single-patient rooms and improved cleaning practices.

Active surveillance for CRAB aims to uncover the hidden pool of CRAB carriers, often likened to the “hidden iceberg effect,” where many individuals carrying CRAB go unnoticed until active screening is employed [26]. Our recent research has also shown that environmental contamination surrounding CRAB carriers is not lower for colonized patients than for patients with clinical infection [27]. Therefore, identifying and isolating carriers appears to be an important strategy. This study underscores the importance of CRAB screening in high-risk units (eg, general ICU) and high-risk settings (eg, multipatient step-up rooms). We might have underestimated the true impact of active screening, as the body sites we sampled and methods we used during the study period for detecting carriers have limited sensitivity [28]. Further investigations employing more sensitive screening techniques are warranted.

Our multimodal intervention incorporated several components recognized as essential for effective infection prevention. These components included the institution management's commitment to infection prevention, active engagement of champions, structured auditing with constructive feedback, and interdisciplinary prevention programs to facilitate behavioral change [29]. In our study, the impact of this intervention was notably modest in settings where infrastructure improvements and staff engagement were already in place (such as the general ICU). However, the intervention appeared to have an impact on HO-CRAB infections in medical wards: The slope became negative, and although the change in slope did not reach statistical significance, this may be because of the limited number of observations.

This success in curtailing CRAB hospital-wide in a high-incidence setting provides support to the view that “endemicity” of CRAB in a hospital is actually a state of multiple simultaneous outbreaks [14] and that confronting each of these outbreaks by unit-specific measures may change the overall epidemiology and lead to a sustained reduction in CRAB incidence. Thus, with appropriate measures taken, CRAB “endemicity” is reversible, and this should be the aim where high endemicity is observed.

The study exhibits several strengths. First, it featured a comprehensive hospital-wide follow-up spanning 14 years. Additionally, it relied on clinical cultures and blood cultures, which are robust outcomes that, unlike detection of asymptomatic carriage, are not influenced by factors such as policy and adherence. Third, we used ITSA, which is a robust method to analyze the impact of interventions.

The study also has some weaknesses. First, as in all observational studies, we cannot prove that the interventions directly caused the observed changes in infection rates. Other unmeasured changes over the long study period, such as changes in case mix or in circulating strains of CRAB, might have contributed to the change in CRAB epidemiology. A stepped wedge cluster randomized trial would be the best design to demonstrate a causal relationship between CRAB prevention interventions and CRAB infection rates. Second, the timing and overlap of several interventions pose a challenge in isolating the specific impact of each intervention. Moreover, the high number of intervention points leads to fewer observations between points, reducing the statistical power in the ITSA. Third, there may have been a halo effect, wherein interventions in 1 ward or department spill over into adjacent units. Lastly, as we have shown, setting-specific variables determine the success of the interventions. Therefore, our findings may not be generalizable to all settings.

###  

In conclusion, a uniform approach is not adequate in the battle against CRAB infections; interventions should be tailored to the specific requirements of diverse patient groups and units. CRAB hyperendemicity should be viewed as a potentially reversible situation.
